# Resignation of Prof. Hare

**Published:** 1847-07

**Authors:** 


*Resignation of Prof. Hare.*—Prof. Hare the distinguished chemist, who for so many years has occupied the chair of Chemistry in the University of Pennsylvania, has resently resigned his professorship. We have not, as yet, learned who is to be his successor.

